# LC-QTOF-MS Analysis and Activity Profiles of Popular Antioxidant Dietary Supplements in Terms of Quality Control

**DOI:** 10.1155/2017/8692516

**Published:** 2017-05-31

**Authors:** Elwira Sieniawska, Tomasz Baj, Rafal Sawicki, Aleksandra Wanat, Krzysztof Kamil Wojtanowski, Grazyna Ginalska, Grazyna Zgorka, Jolanta Szymanska

**Affiliations:** ^1^Chair and Department of Pharmacognosy with Medicinal Plant Unit, Medical University of Lublin, Chodzki 1, 20-093 Lublin, Poland; ^2^Chair and Department of Biochemistry and Biotechnology, Medical University of Lublin, Chodzki 1, 20-093 Lublin, Poland; ^3^Chair and Department of Paedodontics, Medical University of Lublin, Karmelicka 7, 20-081 Lublin, Poland

## Abstract

The dietary supplements with claimed antioxidant activity constitute a substantial part of the dietary supplement market. In this study, we performed the LC-QTOF-MS analysis and investigated the activity profiles of popular antioxidant dietary supplements from different chemical groups in terms of quality control. The commonly used antioxidant tests and statistical analysis revealed that substantial part of the results was comparable if 1 g sample was considered, but while comparing single and daily doses, significant differences in antioxidant values were noticed in all assays. The best antioxidant activity was obtained in ORAC assay (from 142 to 13814 *μ*M of Trolox equivalents per 1 g of sample), and the strongest correlation occurred between TPC and ORAC. The LC-QTOF-MS analysis revealed that catechins were present in samples having the best antioxidant activity and that dietary supplements showing the weakest activity contained very small amount of any chemical constituents.

## 1. Introduction

Dietary supplements are nowadays a big branch of food industry, and their consumption was reported to increase in the recent years [1]. They are claimed to provide health benefits and prevent numerous chronic diseases. Dietary supplements containing antioxidants (vitamins, glutathione, selenium, and plant polyphenols) are recommended to diminish oxidative stress occurring in the human body and causing diseases such as atherosclerosis, arthritis, cancer, cardiovascular disease, and inflammation [1, 2]. Because of the growing interest in natural antioxidants, it is possible to find in the market more and more new products containing mainly plant extracts, their blends, isolated plant secondary metabolites, or algae with declared antioxidant properties. The diversity of active compounds is very large; however, most of them belong to polyphenols. The antioxidant assays comparing different foods, beverages, or herbs use 1 g or 100 g of sample as a unit of measure. However, for finished products (available as dietary supplements), antioxidant activity should be rather described for a single dose (one tablet/capsule) or recommended by a producer daily dose (number of tablets/capsules taken per day). This new approach enables the proper comparison of antiradical power of dietary supplements used to enrich diet in antioxidants. The assessment of activity of a dose taken is important in dietary supplement industry because it also enables to guarantee a safety of supplemented compounds.

In this paper, we aimed to characterize dietary supplements with claimed antiradical activity containing compounds from different chemical groups (anthocyanins, biflavonoids, catechins, curcuminoids, flavonoids, phenolic acids, phlorotannins, proanthocyanidins, and stilbenoids). The performed commonly used antioxidant tests (ORAC, ABTS, and DPPH) as well as the measurement of total phenolics with Folin–Ciocalteu reagent enabled to compare for the first time the activity of single dose and daily dose of each formulation and to draw the correlations between tests used. In terms of quality control, the LC-QTOF-MS analysis was used for a chemical characterization of the studied dietary supplements.

## 2. Experimental

### 2.1. Sample Preparation

All dietary supplements (DS) were commercially available and were bought in the pharmacy store. The active ingredients declared by the manufacturer along with recommended daily dose are listed in the Table 1. Ten capsules or tablets were taken from each package of examined DS. The content of the capsules was quantitatively transferred into the conical flask, whereas tablets were placed in a flask without grinding. Bidistilled water (100 mL) was added to the flask, and the content of the vessel was heated for 1 hour at 37°C and mixed using a magnetic stirrer. Then, the mixture was centrifuged at a speed of 3300 rpm for 10 min, and precipitate was removed by filtration. The obtained aqueous filtrate was freeze-dried (Freeze Dryer, Christ Alpha 2–4 LD, Germany). A similar procedure was repeated for each DS. The lyophilisates were weighed and stored in a vacuum-sealed containers until used.

### 2.2. Determination of Antioxidant Activity

The oxygen radical absorbance capacity assay (ORAC) was adopted from [3], and the *α*,*α*-diphenyl-*β*-picrylhydrazyl (DPPH) assay was adopted from [4]. 2,2′-Azino-bis(3-ethylbenzothiazoline-6-sulphonic acid) (ABTS) assay followed the method of [5] with modifications for microscale measurements. All reactions were done in 3 replicates for each sample (standard and DS samples). Trolox used in the abovementioned tests was dissolved in methanol and diluted to obtain the following concentrations: 3.125, 6.25, 12.0, 25.0, 50.0, 100.0, and 200.0 *μ*M. The samples of DS were prepared by dissolution of exactly weighted 1 mg of each lyophilisate in deionized water, and the dilutions of obtained stock solutions were in the range from 0.01 to 0.1 mg/mL depending on the sample activity. The results obtained for ORAC, DPPH, and ABTS assays were expressed as Trolox equivalents per 1 g of sample, per single dose, and per daily dose of investigated dietary supplements.

The total phenolic content assay (TPC) was adopted from [6] with modifications for microscale measurements. The working solution of Folin–Ciocalteu reagent (FCR) was prepared by 1 : 10 (*v*/*v*) dilution of the stock solution with deionized water. The caffeic acid was dissolved in deionized water and diluted to obtain the following concentrations: 0.625, 1.25, 2.5, 5.0, 10.0, 20.0, and 40.0 *μ*g/mL. The samples of DS were prepared by dissolution of exactly weighted 1 mg of each lyophilisate in deionized water to obtain concentrations in the range from 2.0 to 0.5 mg/mL depending on the sample activity. The experimental wells in the 96-well plate were filled with 10 *μ*L of samples or caffeic acid concentrations, whereas the blank wells received 10 *μ*L of deionized water. Then, 50 *μ*L of FCR was added to every well, and after 30 seconds, all wells were filled with 150 *μ*L of saturated sodium carbonate solution. The plate was incubated in the 40°C for 30 minutes. Next, the absorbance was measured at 740 nm using the microplate reader. The plate reader was controlled by Gen5™ Data Analysis software, which was used also to obtain standard curve by plotting the absorbance of caffeic acid against its various concentrations. All reactions were done in 3 replicates for each sample (standard and antioxidant samples). The results obtained for TPC assay were expressed as caffeic acid equivalents per 1 g of sample, per single dose, and per daily dose of investigated DS.

### 2.3. LC-QTOF-MS Analysis

The LC-QTOF-MS analysis was performed on Agilent 1200 Infinity HPLC chromatograph hyphenated with QTOF 6530B Accurate-Mass QTOF LC/MS system equipped with Dual Agilent Jet Stream spray source (ESI) (Agilent Technologies, Santa Clara, CA, USA) connected with N2 generator (Parker Hannifin Corporation, Haverhill, MA; generating N2 at purities > 99%). HPLC was performed on Gemini® 3 *μ*m i.d. C18 with TMS endcapping, 110 Å, LC Column 100 × 2 mm, and mobile phase was a gradient of 5% acetonitrile in water (A) and 95% acetonitrile in water (B); both phases have a pH value of 4.5 with addition of 10 mM of ammonium formate. A mobile phase gradient was optimized according to the polarity of compounds present in the samples. Samples S1, S10, and S14 were analyzed in the gradients 0–45 min from 5 to 60% of B, 46–55 min from 61 to 90% of B, and 56–60 min 90% of B. For samples S3–S7, S9, S13, and S15, the gradients 0–45 min from 5 to 40% of B, 46–55 min from 41 to 80% of B, and 56–60 min from 81 to 90% of B were applied. Samples S2, S8, S11–12, and S16 were separated in the gradients 0–45 min from 0 to 20% of B, 46–55 min from 21 to 60% of B, and 56–60 min from 61 to 90% of B. The flow rate was 0.1 mL/min. Total analysis time was 75 min. ESI-QTOF-MS analysis was performed in 2 GHz extended dynamic range, negative ionization mode, with fragmentor energy of 160 V, drying gas temp: 350°C, drying gas flow: 12 l/min, sheath gas temp: 400 °C, sheath gas flow: 12 l/min; nebulizer pressure: 35 psig, capillary V (+): 4000 V, and skimmer: 65 V. The acquisition parameters are as follows: auto MS/MS mode; mass range 50–1000 amu for MS and 50–1000 amu for MS/MS experiment; and 1 spectra/s acquisition. The collision-induced dissociation (CID) energy was optimized in the range 10–40 V. The identification of compounds present in samples was performed by comparison of MS/MS spectra with literature data and records from METLIN database. The tentative identification of some derivatives was based on the fragmentation patterns of known compounds.

### 2.4. Statistical Analysis

Each antioxidant activity assay was done in 3 replicates from the same sample in order to determine the precision of method used. The arithmetic mean and median, standard deviation, and coefficient of variation were calculated for the results obtained by various methods. Correlations among data obtained using different assays were calculated using Pearson's correlation coefficient. Statistical calculations were performed using the Statistica software version 10 (StatSoft Inc., Tulsa, OK, USA).

## 3. Results and Discussion

Dietary supplements are a big part of food and pharmaceutical market worldwide. They do not undergo quality controls; hence, sometimes the obtained activity may be different than expected. In this study, dietary supplements with claimed antioxidant activity were tested to check their real value. Preparations consisted of substances from different chemical groups (Table 1) (catechins, curcuminoids, flavonoids, phenolic acids, phlorotannins, proanthocyanidins, and stilbenoids) and showed both very high and negligible antioxidant activities. The water extracts used in this study were prepared during one hour stirring of dietary supplement content in water in 37°C. These conditions were applied to simulate the release from formulation matrix of the compounds soluble in gastrointestinal fluids.

**Figure 1:**
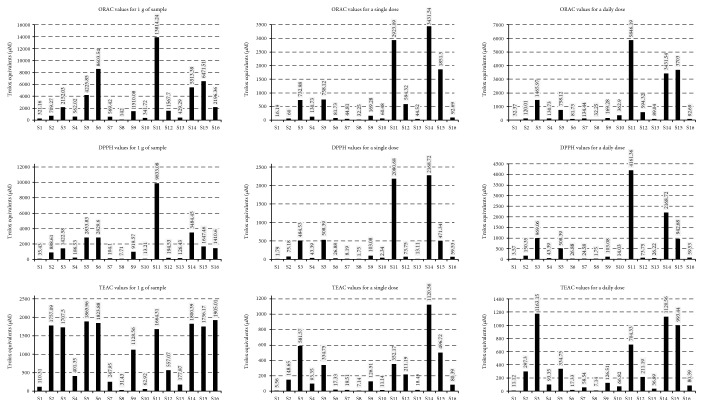
The results obtained for antioxidant capacity assays (ORAC, DPPH, and ABTS) expressed as Trolox equivalents per 1 g of sample, per single dose, and per daily dose of investigated dietary supplements.

**Figure 2:**
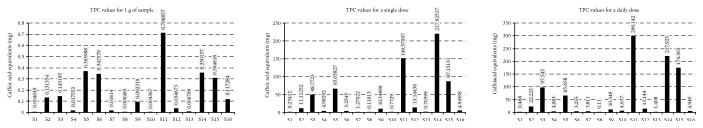
The results obtained for total polyphenol content (TPC) expressed as caffeic acid equivalents per 1 g of sample, per single dose, and per daily dose of investigated dietary supplements.

The results obtained in activity assays (Figures 1 and 2) indicated that the activity of standard unit of measure (1 g/100 g) does not correspond to the activity of the dose recommended for diet supplementation. The highest discrepancies among the doses were noticed for ABTS assay, where substantial part of the results was comparable if we take into account the 1 g sample, but totally different pattern was visible for single and daily doses. Smaller differences were observed for ORAC and DPPH assays, where the best activities were obtained for the same samples. S14 gave the best result as a single dose, but S11 was the most active as a daily dose (with exception of ABTS assay).

**Figure 3:**
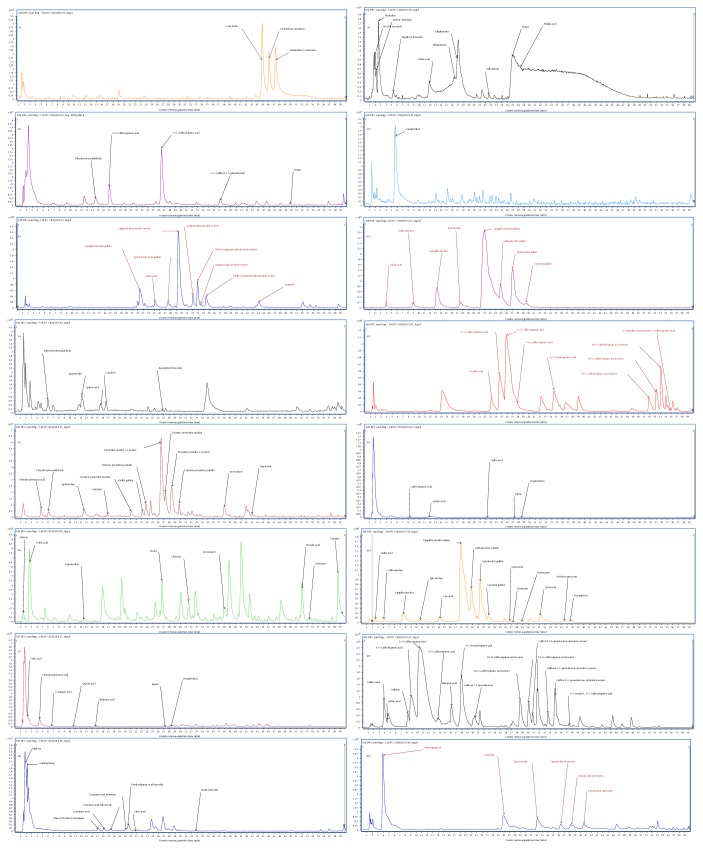
The chemical profiles of studied dietary supplements.

Catechins were the major compounds in the samples which showed the best antioxidant activity (S11 and S14). The supplement with an extract of green tea (S11) contained mainly epi- and gallocatechin gallate as well as epi- and catechin gallate. The ORAC value obtained for S11 (13814.2 *μ*mol Trolox/g) corresponds to the highest result obtained by Seeram et al. who in their study examined supplements with green tea and described results in the range from 218.7 to 13690.7 *μ*mol Trolox/g [7]. It also should be noted that the antioxidant activity of green tea supplement measured in the ORAC assay was 1.4 times greater than that measured in the DPPH assay and up to 8.3 times higher than that measured in the ABTS method, which can be explained by the different reaction mechanisms of catechins and used reagents. This observation is consistent with the results obtained by Tabart et al. who showed that flavan-3-ols such as catechins, epicatechins, and epigallocatechins give a much higher antioxidant activity values in the ORAC method than in ABTS and DPPH assays [8]. The second potent preparation was S14 (OXXYNNEA® and blend of extracts). According to the manufacturer, the preparation should contain grape seed extract (95% of proanthocyanidins), extract from green tea leaves (55% of EGCG), citrus bioflavonoids 40%, extract of *Rhodiola rosea* root (4% of rosavins), extract from the leaves of artichoke (5% of cynarin), extract of cranberry fruit (10% of proanthocyanidins), trans-resveratrol, quercetin, alpha-lipoic acid, coenzyme Q10, astaxanthin, and lycopene. The performed LC-QTOF-MS analysis (Figure 3(), Table S2 available online at https://doi.org/10.1155/2017/8692516) confirmed the presence of catechins, with epigallocatechin gallate as a predominant compound followed by gallocatechin gallate and epicatechin gallate. The other detected compounds were gallic acid, flavonoids, and resveratrol. The proanthocyanidins, rosavins, and cynarin were not detected. From the chemical profile of this sample, it can be seen that catechins are responsible for the quite high antioxidant value obtained in the ORAC assay (5513.39 *μ*mol Trolox/g), but the declared content of preparation was not assured. The sample following catechins in activity was S15. Between the two supplements with green coffee extract, only S15 exerted quite high antioxidant activity and this was consistent with total phenolic content which reached 304.62 and 34.67 mg of caffeic acid equivalents/g for S15 and S12, respectively. Based on these data, it can be concluded that the tested supplements of green tea had significantly different quality in terms of quantity of active compounds in formulation, because the chemical profile of both samples was quite similar with 5-O-caffeoylquinic acid as the main component, followed by other caffeoylquinic acids, 5-O-feruloylquinic acid, dicaffeoylquinic acids, and their derivatives. High antioxidant activity was also showed by extract from the skins and seeds of grapes (S5) and extract from the root of Japanese knotweed (S6). According to the manufacturer's declaration, the main ingredient of these preparations should be resveratrol, and the LC-QTOF-MS analysis revealed the presence of resveratrol; however, in S5, the main compounds were proanthocyanidins A and A-type trimeric proanthocyanidins, while gallic acid, piceid, emodic acid, and emodin were among the major compounds of S6 (Table S2). Although these two supplements were proven to be good antioxidants in terms of 1 g of sample, the daily dose was too low and these samples were placed among the weakest preparations in terms of daily dose activity. Formulations containing goji berry extract (S8), acai berry extract (S4), and powdered fruits of Chinese magnolia (*Schisandra chinensis*) (S7) were proved to be weak antioxidants; however, due to their high nutritional value and high content of polyphenols, they are so-called “superfruits” [9]. Although the literature data refer to high ORAC values for the mentioned fruits, the results obtained in our studies were in the range from 142 to 569.42 *μ*mol Trolox/g, which is in agreement with the studies performed by Henning et al., which showed very low antioxidant values for supplements with acai and goji fruits [10]. The possible reason of low antioxidant activity of these preparations is that the producers use substances of poor quality, or that during treatment process, a loss of active ingredients occurs, because among the identified compounds in S4 were only catechins and quinic acid, S7 contained organic and phenolic acids, while in S8, cabrohydrates and phenolic glycosides were present. Supplement with another “superfruit,” a pomegranate in the form of an extract, showed moderate antioxidant activity at the level of 1510.08 *μ*mol Trolox/g in ORAC assay. This result was placed in the middle comparing to results obtained by Madrigal-Carballo et al. in an ORAC method [11]. Madrigal-Carballo et al. evaluated supplements with pomegranate, which showed antioxidant activity values between 59 and 3210 *μ*mol Trolox/g with an average of 1127 *μ*mol Trolox/g. As was reported, supplements with pomegranate are often falsified, which can be detected by analyzing the qualitative composition of polyphenol fraction. True skin extract and pomegranate seeds contain a high amount of punicalagin, punicalin, and gallo- and elagotanins [11]. The LC-QTOF-MS analysis performed in our study confirmed the authenticity of the extract (Table S2). The lack of punicalagin may be explained by used scan range which was between 50 and 1000 *m*/*z*. Low antioxidant activity results were obtained for preparations with powdered rhizome of turmeric (S1) and powdered fruits of hawthorn (S13), although these substances are considered to be valuable antioxidants. According to Wu et al., the total antioxidant capacity of 1 g of powdered rhizome of turmeric is 1592.77 *μ*mol Trolox/g [12]. It is the sum of the values obtained for lipophilic (lipophilic ORAC (L-ORAC)) and hydrophilic compounds (hydrophilic ORAC (H-ORAC)), which are 1193.46 *μ*mol Trolox/g (L-ORAC) and 399.31 *μ*mol Trolox/g (H-ORAC) [12]. The high value of L-ORAC confirms that compounds responsible for antioxidant properties of curcumin: curcuminoids and volatile oil, are contained in the lipophilic fraction [13] and that curcuminoids detected in the studied water extract had to lower concentration to exert a potent antioxidant effect (321.16 *μ*mol Trolox/g). According to the data obtained by Kratchanova et al. also, antioxidants of the hawthorn fruit are lipophilic [14]. The ORAC value described by these authors for an aqueous hawthorn extract was 364 *μ*mol Trolox/g, whereas 2163 *μ*mol Trolox/g for acetone hawthorn extract [14]. In our study, the chromatographic profile of the aqueous extract of supplement containing hawthorn was poor explaining the low antioxidant activity (428.29 *μ*mol Trolox/g). Two of the tested supplements contained ingredients different than plants. Supplement S10 contained spirulina or powdered fronds of freshwater algae, and supplement S16 contained an extract from brown algae *Ecklonia cava*. Despite the antioxidant properties of spirulina, proven by Abd El-Baky et al. [15] in our study, formulation with spirulina had the weakest antioxidant activity and the LC-QTOF-MS analysis confirmed poor extract composition with main, unidentified compound having parent ion at 218 *m*/*z* (M-H)^−^. Preparation with *Ecklonia cava* showed moderate antioxidant activity and interestingly gave the highest result in the ABTS method suggesting good affinity of detected phloroglucinol to ABTS reagent. It also should be noted that despite many scientific reports on the antioxidant activity of these algae, as well as other species of freshwater and saltwater algae, the antioxidant properties of these species are much less frequently investigated than activity of higher plants.

**Figure 4:**
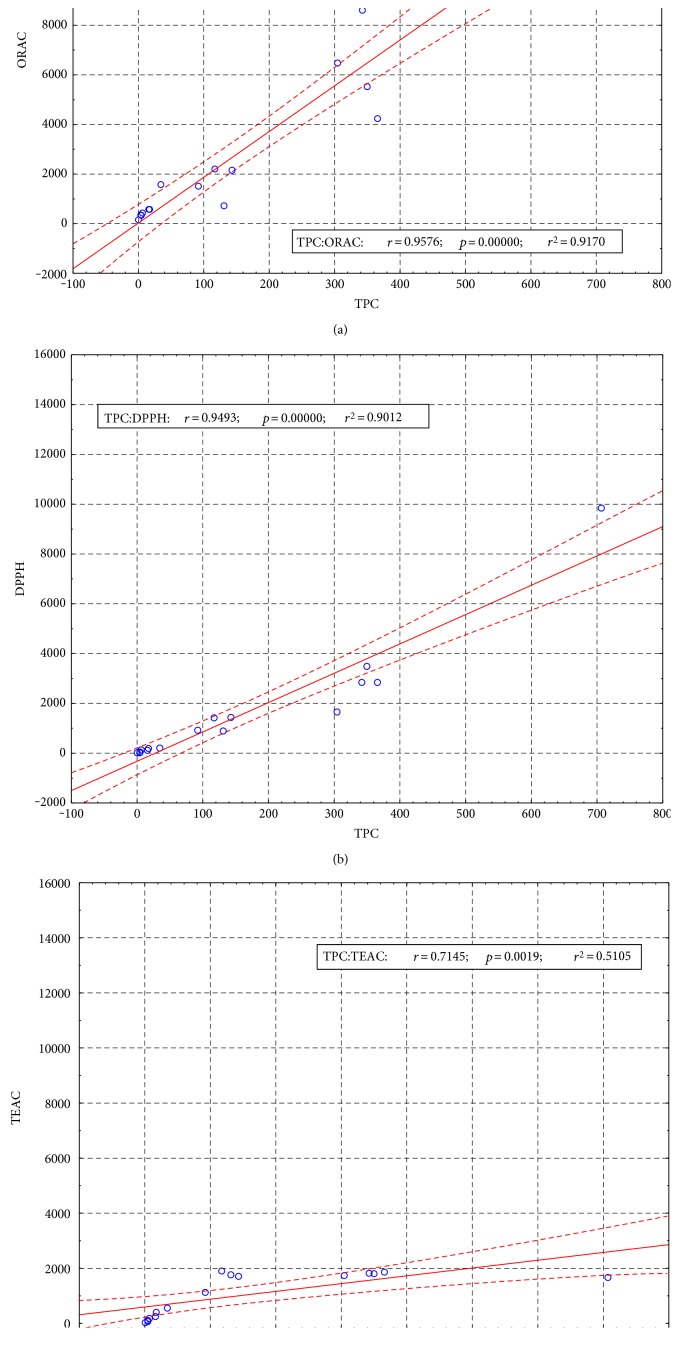
The Pearson's correlations between results obtained using different antioxidant activity tests and total polyphenol content (TPC). (a) ORAC versus TPC, (b) DPPH versus TPC, and (c) ABTS versus TPC.

The results obtained in different assays ranged several orders. The ORAC value for sample S10 was 341.72 *μ*mol Trolox/g, whereas the same sample gave only 13.21 *μ*mol Trolox/g in the DPPH test. Similar differences were shown for S11: 13814.24 and 1664.31 *μ*mol Trolox/g were obtained in ORAC and ABTS assays, respectively. This confirms that each method for antioxidant activity testing is specific and in some terms imperfect. Despite the use of Trolox as a universal reference and calculating the results on the Trolox equivalents, comparing the results obtained using different methods may lead to different conclusions. Antioxidants react differently with reagents used in the determination of the antioxidant capacity, and this gives inconsistent results. Our findings are supported by the literature data. In the study conducted by Zulueta et al., antioxidant activity of ascorbic acid was twice higher in ABTS than in the ORAC method although the results obtained for gallic acid were comparable in both methods [16]. Tabart et al. compared antioxidant activity of 22 various phenolic compounds by three methods: ABTS, DPPH, and ORAC. Significant differences in the results expressed as Trolox equivalents were observed for 16 samples in ABTS, for 11 in DPPH, and for 21 in ORAC method [8]. In addition, the antioxidant activity measured by ORAC was the highest and that measured by DPPH was the lowest [8]. A similar relationship exists also between our results. The arithmetic mean of the different methods gave the highest average value in the ORAC method, while the lowest in ABTS, but calculated median value was highest in the ORAC method and lowest in the DPPH method. The discrepancies between the various methods may be caused by the different physicochemical properties of the reagents (including standards) and samples (the color, dispersion), the mechanism of reaction of the reagents with the tested compounds, and assay conditions—the type of solvent, pH, temperature, time of the measurement, and the type of measuring apparatus [17]. Depending on the structure of the antioxidants tested, the reaction with the reagent may require different time. ABTS reacts with the antioxidants immediately, in less than mixing time, for example, with Trolox or chlorogenic acid. However, some antioxidants do not react with ABTS or react slowly in low concentrations of a more rapidly at higher concentrations; this includes quercetin and curcumin [17]. The reaction of ABTS and DPPH radicals with the sample components is difficult because of their steric hindrance and difficult access to the atom with an unpaired electron. The reaction with DPPH is also very sensitive to the type of solvent, pH, oxygen, and light. Furthermore, these methods measure only the end result of the reaction, neglecting the kinetics and the effect of antioxidant concentration [17]. It appears that the most reliable method of determining antioxidant activity is the ORAC method because the measurements are based on the continuous generation of radicals in real time, as it occurs in the living body [2]. The specificity of the methods also affects the correlations between the results obtained from different methods. It is difficult to clearly define the strength of the correlations because, depending on the sample matrix, the type of antioxidants, and their physicochemical properties, the correlation between various methods can be very strong or may not exist at all. The samples examined in this study were not uniform in terms of quality, formulation, and chemical composition; however, statistically significant correlations between the different methods were observed (Table 2, Figure 4()). The strongest correlation occurred between the ORAC and DPPH tests, while the weakest correlations with the other assays gave ABTS method. Based on the calculated correlation coefficients, it can also be said that there were a strong correlation between the antioxidant activity and total polyphenol content measured by the Folin–Ciocalteu assay. The strongest correlation of TFC occurred with ORAC results while the weakest with ABTS. The literature data are partially in opposition with the obtained results. Stintzing et al. who studied the antioxidant activity of prickly pear juice demonstrated a strong correlation between the results obtained by ORAC and using ABTS (*r* = 0.974) [18], while Silva et al. showed only a weak correlation (*r* = 0.551) between results of these antioxidant tests conducted for 15 plants grown in Brazil [19]. In turn, the study of white and red wines did not show any significant correlation between the ORAC, TRAP, and ABTS methods [20] and no correlation was found for the results of antioxidant capacity of human plasma tested using ORAC and ABTS assays [21]. Murillo and coworkers studied 39 exotic fruits for the content of polyphenolic compounds and their antioxidant properties. The correlation between the results of the ABTS and the TPC in the tested fruits was *r* = 0.89 [22]. On the other hand, research on the species of medicinal plants from Nepal showed a weak correlation between phenolic content and the inhibition of DPPH (*r* = 0.3004) [23]. The described literature data demonstrate that it is not possible to compare different antioxidant assays because of the different chemistries involved with the different methods. Also, the requirement for equivalency between assays was revised and removed [24].

**Figure 5:**
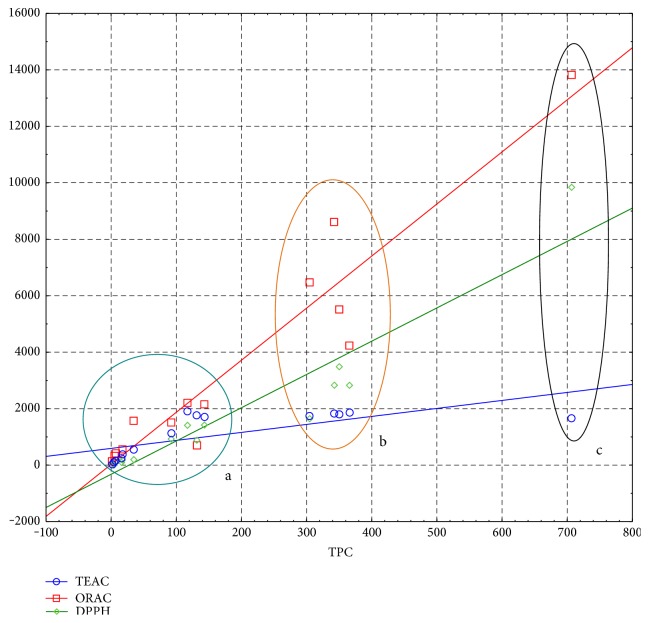
The scatterplots showing correlation between TPC and activity (ABTS, ORAC, and ABTS) and the number of supplement. (a) S1-S4, S7-S10, and S12-S13; (b) S5-S6 and S14-S15; and (c) S11.

The above-discussed activity in correlation with sample chemical composition is in agreement with scatterplots describing the correlation between TPC and the type of DS (Figure 5). The highest activity and TPC value for a daily dose obtained for green tea placed this supplement as a separate group on the cluster graph indicating good quality and high antioxidant potential of tested preparation. In the second group, DS containing catechins, proanthocyanidines, resveratrol, and caffeoylquinic acids were found showing their quite good quality. The remaining preparations formed the biggest cluster characterized by the weakest activity and the very poor composition in some cases. These results prove that because quality control is not applied during the production of dietary supplements, many of the preparations investigated in this study are of uncertain quality. The desired antioxidant effect may not be obtained in the case of these samples because the single dose and the daily dose contain small amount of compounds exerting antioxidant activity. Currently, in many countries, the status of dietary supplements is discussed. These products are popular and used among people of all ages. Some countries seek to restrict the sale of supplements in pharmacies and directing them mainly to shopping areas. However, it is worth to remember that dietary supplement is not only a food but also a mixture of a number of biologically active components that have an impact on our body. Therefore, a greater supervision over the composition of these kinds of products is necessary, and the manufacturers should be more responsible when declaring their qualitative and quantitative composition.

## 4. Conclusions

In this study, we showed that while investigating the functional products such as preparations of dietary supplements, the criterion of a single and a daily dose should not be omitted. The results obtained in activity assays and calculated on the standard unit of measure (1 g/100 g) do not correspond to the activity of the dose suggested for diet supplementation. A substantial part of the results is comparable if we take into account the 1 g sample, while comparing single and daily doses, significant differences in the antioxidant value of preparations are noticed in all assays. The highest antioxidant values were obtained in the ORAC assay, and the strongest correlation occurred between total phenol content and ORAC results suggesting that the most reliable method of determining antioxidant activity is ORAC. The best antioxidant activity was obtained for preparations containing catechins, suggesting these compounds may be responsible for a beneficial effect of dietary supplements. The performed analyses revealed that the majority of studied dietary supplements contain pharmacologically active ingredients. However, some products had poor quality, resulting in the need to increase the number of doses taken. Consumers who purchase dietary supplements to maintain a good health have no knowledge of the effective dose. Hence, the whole responsibility for the therapeutic effect of dietary supplement lies on the manufacturer who should assure the safety and quality of the product.

## Supplementary Material

Table S1. Antioxidant activity of dietary supplements. Table S2. The chemical composition of investigated dietary supplements.

## Figures and Tables

**Table 1:** The declared ingredients of investigated dietary supplements.

	Composition of the supplement (1 capsule/tablet)		Formulation	Recommended daily dose
	Ingredients^∗^	Mass [mg]		
S1 (turmeric)	Turmeric rhizome powder	720	Capsule	2

S2 (bilberry)	Powdered bilberry fruit	250	Tablet	2
	Extract of bilberry including the following:	40		
	(i) Anthocyanins	10		
	(ii) Vit. C	40		
	(iii) Vit. E	6		
	(iv) Lutein	3		
	(v) Beta carotene	83.5		

S3 (blend of extracts)	Extract from the root of Baikal skullcap	150	Capsule	2
	Powdered cinnamon bark	60		
	Cranberry fruit extract	50		
	Extract from green tea leaves	50		
	Extract of the herb of horsetail	45		
	Vit. C	40		
	Extract of chokeberry	20		
	Extract from rhizomes of ginger	12		
	Extract of bilberry fruit	10		
	Extract of grape fruit	3		

S4 (acai)	Extract of acai berry	300	Capsule	1

S5 (grapes)	Grape skin extract including	400	Capsule	1
	(i) Trans-resveratrol	200		
	Grape seed extract including	100		
	(i) Proanthocyanidins	95		

S6 (resveratrol)	Resveratrol (from an extract of *Reynoutria japonica*)	50	Capsule	1

S7 (*Schisandra*)	Powdered fruits of the *Schisandra chinensis*	525	Capsule	

S8 (goji)	Extract of goji fruit including	300	Capsule	1
	(i) Polysaccharides	150		

S9 (pomegranate)	Pomegranate peel extract including	300	Capsule	1
	(i) Elagic acid	120		

S10 (spirulin)	Spirulin powder	450	Capsule	3–6

S11 (green tea)	55% green tea extract including the following:	250	Capsule	1-2
	(i) EGCG (epigallocatechin gallate)	137.5		
	(ii) Polyphenols	249		
	(iii) Catechins	200		

S12 (green coffee)	Extract of green coffee including the following:	800	Capsule	1
	(i) Caffeine	34.8		
	(ii) Chlorogenic acid	400		

S13 (hawthorn)	Powdered fruit of hawthorn	565	Capsule	1-2

S14 (OXXYNNEA and blend of extracts)	OXXYNNEA^∗∗^	200	Capsule	1
	Grape seed extract (95% of proanthocyanidins)	150		
	Extract from green tea leaves (55% of EGCG)	150		
	Citrus bioflavonoids 40%	150		
	Trans-resveratrol	100		
	Extract of *Rhodiola rosea* root (4% of rosavins)	100		
	Quercetin	100		
	Extract from the leaves of artichoke (5% of cynarin)	50		
	Extract of cranberry fruit (10% of proanthocyanidins)	40		
	Alpha-lipoic acid	30		
	Coenzyme Q10	15		
	Astaxanthin	5		
	Lycopene	1		
	Beta carotene	1		

S15 (green coffee)	An extract of green coffee beans (50% of chlorogenic acid) including	400	Capsule	2
	(i) Caffeine	20		

S16 (Ecklonia)	*Ecklonia cava* extract 25 : 1 (98.8% pure *Ecklonia cava*—the stem and leaves standardized to 15% polyphenols and phlorotannins)	53	Capsule	1

^∗^The fillers and additives forming capsule/tablet were omitted; ^∗∗^OXXYNNEA—the blend of extracts of fruits and vegetables: white and red grapes, oranges, grapefruit, blueberry, papaya, pineapple, strawberries, apples, apricots, cherries, black currants, tomato, carrot, green tea, broccoli, cabbage, onions, garlic oil, wheat germ, cucumber, and asparagus.

**Table 2:** Values of Pearson's *r* correlation coefficients for the antioxidant assays used.

	ABTS	ORAC	DPPH	TPC
ABTS	—	0.6142	0.5619	0.7145
ORAC	0.6142	—	0.9254	0.9576
DPPH	0.5619	0.9254	—	0.9493
TPC	0.7145	0.9576	0.9493	—

All results were statistically significant for *p* < 0.05.
