# Supplementary material for: LC-QTOF-MS Analysis and Activity Profiles of Popular Antioxidant Dietary Supplements in Terms of Quality Control
Source: Oxid Med Cell Longev. 2017 May 31;2017:8692516. doi: 10.1155/2017/8692516 (PMC5470020; doi:10.1155/2017/8692516)
Supplement: Supplementary file 1 — Table S1. Antioxidant activity of dietary supplements. Table S2. The chemical composition of investigated dietary supplements. [file 8692516.f1.doc]

**Supplementary material**

**Table S1. Antioxidant activity of dietary supplements.**

|  | **ORAC (Trolox equivalents (μM))** | | | **DPPH (Trolox equivalents (μM))** | | | **ABTS (Trolox equivalents (μM))** | | | **TPC (caffeic acid equivalents (mg))** | | |
| --- | --- | --- | --- | --- | --- | --- | --- | --- | --- | --- | --- | --- |
|  | 1g of sample | Single dose | Dailydose | 1g of sample | Single dose | Dailydose | 1g of sample | Single dose | Dailydose | 1g of sample | Single dose | Dailydose |
| **S1** | 321.16 | 16.19 | 32.37 | 35.43 | 1.79 | 3.57 | 110.31 | 5.56 | 11.12 | 0.004645 | 0.23412 | 0.468 |
| **S2** | 709.27 | 60 | 120.01 | 888.61 | 75.18 | 150.35 | 1757.09 | 148.65 | 297.3 | 0.131354 | 11.11252 | 22.225 |
| **S3** | 2152.03 | 732.98 | 1465.97 | 1422.58 | 484.53 | 969.06 | 1707.5 | 581.57 | 1163.15 | 0.143195 | 48.7723 | 97.545 |
| **S4** | 562.02 | 130.73 | 130.73 | 186.53 | 43.39 | 43.39 | 401.35 | 93.35 | 93.35 | 0.017553 | 4.08292 | 4.083 |
| **S5** | 4225.85 | 758.12 | 758.12 | 2833.83 | 508.39 | 508.39 | 1865.96 | 334.75 | 334.75 | 0.365988 | 65.65827 | 65.658 |
| **S6** | 8603.54 | 81.73 | 81.73 | 2829.6 | 26.88 | 26.88 | 1823.88 | 17.33 | 17.33 | 0.342579 | 3.2545 | 3.255 |
| **S7** | 569.42 | 44.81 | 134.44 | 104.1 | 8.19 | 24.58 | 247.95 | 19.51 | 58.54 | 0.01614 | 1.27022 | 3.811 |
| **S8** | 142 | 32.25 | 32.25 | 7.71 | 1.75 | 1.75 | 31.43 | 7.14 | 7.14 | 0.000485 | 0.11013 | 0.11 |
| **S9** | 1510.08 | 169.28 | 169.28 | 919.57 | 103.08 | 103.08 | 1128.56 | 126.51 | 126.51 | 0.092319 | 10.34899 | 10.349 |
| **S10** | 341.72 | 60.48 | 362.9 | 13.21 | 2.34 | 14.03 | 62.92 | 11.14 | 66.82 | 0.004367 | 0.7729 | 4.637 |
| **S11** | 13814.24 | 2923.09 | 5846.19 | 9833.08 | 2080.68 | 4161.36 | 1664.31 | 352.17 | 704.33 | 0.706857 | 149.57097 | 299.142 |
| **S12** | 1567.7 | 594.32 | 594.32 | 194.53 | 73.75 | 73.75 | 557.07 | 211.19 | 211.19 | 0.034673 | 13.14436 | 13.144 |
| **S13** | 429.29 | 44.52 | 89.04 | 126.43 | 13.11 | 26.22 | 177.87 | 18.45 | 36.89 | 0.006789 | 0.70399 | 1.408 |
| **S14** | 5513.39 | 3431.54 | 3431.54 | 3484.45 | 2168.72 | 2168.72 | 1800.39 | 1120.56 | 1120.56 | 0.350137 | 217.92517 | 217.925 |
| **S15** | 6471.51 | 1851.5 | 3703 | 1647.46 | 471.34 | 942.68 | 1736.17 | 496.72 | 993.44 | 0.304618 | 87.15131 | 174.303 |
| **S16** | 2196.36 | 92.69 | 92.69 | 1410.6 | 59.53 | 59.53 | 1905.01 | 80.39 | 80.39 | 0.117284 | 4.94938 | 4.949 |

**Table S2. The chemical composition of** investigated dietary supplements

| Sample | Compound | Rt (min) | Molecular formula | [M-H]^-^ (m/z) | Fragment ions | Reference |
| --- | --- | --- | --- | --- | --- | --- |
| S1 | Curcumin | 44.9 | C_21_H_20_O_6_ | 367.1172 | 217.0497; 173,0599; 149.0601; 134.0364 | METLIN (database) |
|  | Demethoxycurcumin | 50.5 | C_20_H_18_O_5_ | 337.1051 | 217.0490; 173.0595; 149.0601; 119.0493 | METLIN (database) |
|  | Bisdemthoxycurcumin | 51.5 | C_19_H_16_O_4_ | 307.0958 | 187.0393; 143.0497; 119.0501 | METLIN (database) |
| S2 | Dihydroxybenzaldehyde | 14.5 | C_7_H_6_O_3_ | 137.0251 | 108.0220 | METLIN (database) |
|  | 3-O-caffeoylquinic acid | 17.0 | C_16_H_18_O_9_ | 353.0863 | 191.0542; 179.0328; 135.0435 | (Clifford MN et al. 2006) |
|  | 5-O-caffeoylquinic acid | 26.2 | C_16_H_18_O_9_ | 353.0808 | 191.0518; 179.0316; 161.0233 | (Clifford MN, Knight S, Surucu B and Kuhnert N 2006) |
|  | 3-O-caffeoyl-1,5-quinolactone | 37.1 | C_16_H_16_O_8_ | 335.0920 | 161.0690; 135.0440 | METLIN (database) |
|  | Rutin | 49.8 | C_27_H_30_O_16_ | 609.1425 | 300.0261 | METLIN (database) |
| S3 | Epigallocatechin gallate | 22.4 | C_22_H_18_O_11_ | 457.0792 | 305.0681; 169.0154; 125.0249 | (Wu C et al. 2012) |
|  | Gallic acid | 25.2 | C_7_H_6_O_5_ | 169.0128 | 125.0232; 79.0188 | (Wu C, Xu H, Héritier J and Andlauer W 2012) |
|  | Epicatechin gallate | 27.4 | C_22_H_18_O_10_ | 441.0886 | 289.0765; 169.0172; 125.0264 | (Wu C, Xu H, Héritier J and Andlauer W 2012) |
|  | Apigenin glucuronide isomer | 29.4 | C_21_H_18_O_1_ | 445.0806 | 269.0488; 175.0296 | (He L et al. 2016) |
|  | Apigenin glucuronide isomer | 32.0 | C_21_H_18_O_1_ | 445.0841 | 269.0514; 175.0282 | (He L, Zhang Z, Lu L, Liu Y, Li S, Wang J, Song Z, Yan Z and Miao J 2016) |
|  | Methoxy apigenin glucuronide isomer | 32.9 | C_22_H_21_O_2_ | 459.1021 | 283.0647; 268.0352; 175.0274 | METLIN (database) |
|  | Apigenin glucuronide isomer | 33.5 | C_21_H_18_O_1_ | 445.0841 | 269.0514; 175.0282 | (He L, Zhang Z, Lu L, Liu Y, Li S, Wang J, Song Z, Yan Z and Miao J 2016) |
|  | Methoxy apigenin glucuronide isomer | 34.5 | C_22_H_21_O_2_ | 459.1021 | 283.0647; 268.0352; 175.0274 | METLIN (database) |
|  | Apigenin | 44.1 | C_15_H_10_O_5_ | 269.0498 | - | (He L, Zhang Z, Lu L, Liu Y, Li S, Wang J, Song Z, Yan Z and Miao J 2016) |
| S4 | Dihydroxybenzaldehyde | 5.9 | C_7_H_6_O_3_ | 137.0251 | 108.0220 | METLIN (database) |
|  | Epicatechin | 12.3 | C_15_H_14_O_6_ | 289.0716 | 245.0823; 205.0500; 125.0239 | (Wu C, Xu H, Héritier J and Andlauer W 2012) |
|  | Quinic acid | 15.5 | C_7_H_12_O_6_ | 191.0590 | 85.0317 | METLIN (database) |
|  | Catechin | 16.5 | C_15_H_14_O_6_ | 289.0712 | 245.0821; 205.0616; 125.0261 | (Wu C, Xu H, Héritier J and Andlauer W 2012) |
|  | Kaempferol hexoside | 26.7 | C_21_H_20_O_11_ | 447.0960 | 285.0447 | METLIN (database) |
| S5 | Dihydroxybenzoic acid | 4.7 | C_7_H_6_O_4_ | 153.0192 | 108.0228; 91.0204; 65.0019 | METLIN (database) |
|  | Dihydroxybenzaldehyde | 6.2 | C_7_H_6_O_3_ | 137.0247 | 108.0222; 92.0266 | METLIN (database) |
|  | Epicatechin | 12.5 | C_15_H_14_O_6_ | 289.0716 | 245.0823; 205.0500; 125.0239 | (Wu C, Xu H, Héritier J and Andlauer W 2012) |
|  | Catechin | 32.1 | C_15_H_14_O_6_ | 289.0712 | 245.0821; 205.0616; 125.0261 | (Wu C, Xu H, Héritier J and Andlauer W 2012) |
|  | A-type procyanidin trimer | 21.0 | C_45_H_36_O_18_ | 863.1839 | 711.1351; 575.1207; 423.0724; 287.0558; 165.0149 | (Qiang L et al. 2015) |
|  | Methyl gallate | 23.1 | C_8_H_8_O_5_ | 183.0313 | 139.0379; 95.0570 | METLIN (database) |
|  | A-type procyanidin trimer | 23.7 | C_45_H_36_O_18_ | 863.1839 | 711.1351; 575.1207; 423.0724; 287.0558; 165.0149 | (Qiang L, Luo F, Zhao X, Liu Y, Hu G, Sun C, Li X and Chen K 2015) |
|  | Proanthocyanidin A isomer | 26.4 | C_30_H_24_O_12_ | 575.1048 | 539.0789; 449.0803; 289.0639; 245.0842; 125.0183 | (Qiang L, Luo F, Zhao X, Liu Y, Hu G, Sun C, Li X and Chen K 2015) |
|  | A-type procyanidin trimer | 27.8 | C_45_H_36_O_18_ | 863.1839 | 711.1351; 575.1207; 423.0724; 287.0558; 165.0149 | (Qiang L, Luo F, Zhao X, Liu Y, Hu G, Sun C, Li X and Chen K 2015) |
|  | Proanthocyanidin A isomer | 28.6 | C_30_H_24_O_12_ | 575.1048 | 539.0789; 449.0803; 289.0639; 245.0842; 125.0183 | (Qiang L, Luo F, Zhao X, Liu Y, Hu G, Sun C, Li X and Chen K 2015) |
|  | A-type procyanidin trimer | 29.9 | C_45_H_36_O_18_ | 863.1839 | 711.1351; 575.1207; 423.0724; 287.0558; 165.0149 | (Qiang L, Luo F, Zhao X, Liu Y, Hu G, Sun C, Li X and Chen K 2015) |
|  | Resveratrol | 37.9 | C_14_H_12_O_3_ | 227.0707 | 185.0595;143.0496 | (Gao F et al. 2016) |
|  | Quercetin | 43.3 | C_15_H_10_O_7_ | 301.0373 | 178.9987; 151.0037 | (Sanchez-Rabaned F et al. 2003) |
| S6 | Hexose | 1.5 | C_6_H_12_O_6_ | 179.0572 | 161.0490; 101.0224; 89.0295 | METLIN (database) |
|  | Gallic acid | 2.6 | C_7_H_6_O_5_ | 169.0128 | 125.0232; 79.0188 | (Wu C, Xu H, Héritier J and Andlauer W 2012) |
|  | Epicatechin | 12.5 | C_15_H_14_O_6_ | 289.0716 | 245.0823; 205.0500; 125.0239 | (Wu C, Xu H, Héritier J and Andlauer W 2012) |
|  | Piceid | 26.6 | C_20_H_22_O_8_ | 389.1261 | 227.0720; 185.0553; 143.0514 | METLIN (database) |
|  | Catechin | 31.7 | C_15_H_14_O_6_ | 289.0712 | 245.0821; 205.0616; 125.0261 | (Wu C, Xu H, Héritier J and Andlauer W 2012) |
|  | Resveratrol | 37.9 | C_14_H_12_O_3_ | 227.0716 | 185.0606; 143.0480 | (Gao F, Zhou T, Hu Y, Lan L, Heyden YV, Crommen J, Lu G and Fan G 2016) |
|  | Resveratrol derivative | 44.3 | C_21_H_30_O_13_ | 489.1588 | 227.0715; 185.0605 | (Gao F, Zhou T, Hu Y, Lan L, Heyden YV, Crommen J, Lu G and Fan G 2016) |
|  | Emodic acid | 52.1 | C_15_H_8_O_7_ | 299.0173 | 255.0307; 227.0351; 211.0388; 183.0346; 155.0506 | METLIN (database) |
|  | Physcion | 53.3 | C_16_H_12_O_5_ | 283.0615 | 268.0337; 240.0411 | (Gao F, Zhou T, Hu Y, Lan L, Heyden YV, Crommen J, Lu G and Fan G 2016) |
|  | Emodin | 58.8 | C_15_H_10_O_5_ | 269.0458 | 240.0425; 225.0548; 210.0303; 197.0602; 182.0365; 171.0442 | (Gao F, Zhou T, Hu Y, Lan L, Heyden YV, Crommen J, Lu G and Fan G 2016) |
| S7 | Citric acid | 1.7 | C_6_H_8_O_7_ | 191.0203 | 111.0087; 87.0103; 67.0216 | METLIN (database) |
|  | Dihydroxybenzoic acid | 4.6 | C_7_H_6_O_4_ | 153.0197 | 108.0222, 91.0204; 65.0019 | METLIN (database) |
|  | Coumaric acid | 6.8 | C_9_H_8_O_3_ | 163.0425 | 119.0481 | METLIN (database) |
|  | Quinic acid | 10.8 | C_7_H_12_O_6_ | 191.0590 | 85.0317 | METLIN (database) |
|  | Shikimic acid | 14.4 | C_7_H_10_O_5_ | 173.0456 | 137.8011; 111.0116; 93.0320 | METLIN (database) |
|  | Rutin | 27.4 | C_27_H_30_O_16_ | 609.1425 | 300.0261 | METLIN (database) |
|  | Isoquercitrin | 28.3 | C_21_H_20_O_12_ | 463.0877 | 300.0238; 271.0247; 255.0295; 243.0284; 151.0051 | (Sanchez-Rabaned F, O., Lamuela-Raventos RM, Bastida J, Viladomat F and Codina C 2003) |
| S8 | Maltose | 1.9 | C_12_H_22_O_11_ | 341.1124 | 221.0693; 179.0583, 161.0478; 143.0371; 119.0367; 101.0264 | METLIN (database) |
|  | Maltohexaose | 2.3 | C_30_H_52_O_26_ | 989.3267 | 827.2736; 665.2195; 503.1656; 341.1123; 161.0477 | METLIN (database) |
|  | Glucosyl hydroxycinnamate | 15.0 | C_15_H_18_O_8_ | 325.0992 | 163.0429; 145.0344 | (Bondia-Pons I et al. 2014) |
|  | Coumaric acid | 15.9 | C_9_H_8_O_3_ | 163.0425 | 119.0481 | (Bondia-Pons I, Savolainen O, Törrönen R, Martinez JA, Poutanen K and Hanhineva K 2014)b |
|  | Coumaric acid dihexoside | 17.3 | C_21_H_28_O_13_ | 487.1506 | 325.0975; 163.0421; 119.0483 | (Bondia-Pons I, Savolainen O, Törrönen R, Martinez JA, Poutanen K and Hanhineva K 2014) |
|  | Coumaric acid hexoside | 20.2 | C_15_H_18_O_8_ | 325.0975 | 163.0423; 119.0482 | (Bondia-Pons I, Savolainen O, Törrönen R, Martinez JA, Poutanen K and Hanhineva K 2014) |
|  | Feruloylquinic acid trihexoside | 20.5 | C_35_H_50_O_24_ | 853.4157 | 691.3594; 529.3096 | (Clifford MN, Knight S, Surucu B and Kuhnert N 2006) |
|  | Citric acid | 21.9 | C_6_H_8_O_7_ | 191.0580 | 111.0102; 87.0117; 67.0168 | (Bondia-Pons I, Savolainen O, Törrönen R, Martinez JA, Poutanen K and Hanhineva K 2014) |
|  | Rutin hexoside | 33.0 | C_33_H_40_O_21_ | 771.2007 | 609.1506; 462.0858; 301.0379 | (Bondia-Pons I, Savolainen O, Törrönen R, Martinez JA, Poutanen K and Hanhineva K 2014) |
| S9 | HHDP-hexoside | 1.9 | C_20_H_18_O_14_ | 481.0655 | 301.0017; 275.0251; 229.0046 | (Brighenti V et al. 2016) |
|  | HHDP-hexoside | 2.2 | C_20_H_18_O_14_ | 481.0637 | 301.0012, 275.0231; 229.0051 | (Brighenti V, Groothuis SF, Prencip FP, Amir R, Benvenuti S and Pellati F 2016) |
|  | Gallagyl-hex (punicalin) | 2.6 | C_34_H_22_O_22_ | 781.0568 | 601.0013; 448.9772 | (Brighenti V, Groothuis SF, Prencip FP, Amir R, Benvenuti S and Pellati F 2016) |
|  | Digalloyl-hexoside | 5.4 | C_20_H_20_O_14_ | 483.0802 | 331.0703; 313.0586; 169.0167 | (Brighenti V, Groothuis SF, Prencip FP, Amir R, Benvenuti S and Pellati F 2016) |
|  | Gallic acid | 11.8 | C_7_H_6_O_5_ | 169.0128 | 125.0232; 79.0188 | (Wu C, Xu H, Héritier J and Andlauer W 2012)Wu 2012 |
|  | Ellagitannin | 16.3 | C_24_H_14_O_15_ | 541.0369 | 301.026; 275.0221 | METLIN (database) |
|  | Ellagitannin | 16.8 | C_27_H_22_O_18_ | 633.0744 | 463.0521; 301.0021, 275.0240 | METLIN (database) |
|  | Gallotannin | 22.6 | C_27_H_24_O_18_ | 635.0903 | 483.0714; 331.0713; 169.0144 | METLIN (database) |
|  | Rutin | 26.9 | C_27_H_30_O_16_ | 609.1425 | 300.0261 | METLIN (database) |
|  | Ellagic acid | 30.1 | C_14_H_6_O_8_ | 300.0029 | 283.9937; 245.0045; 200.0136; 173.0249; 145.0305 | METLIN (database) |
| S10 | Unidentified | 5.7 | C_11_H_11_N_2_O_3_ | 218.0707 | 175.0635; 118.0289; 92.0287 | - |
| S11 | Gallic acid | 4.0 | C_7_H_6_O_5_ | 169.0128 | 125.0232; 79.0188 | (Wu C, Xu H, Héritier J and Andlauer W 2012) |
|  | Gallocatechin | 8.9 | C_15_H_14_O_7_ | 305.0671 | 261,0762; 169.0359; 125.0244 | (Wu C, Xu H, Héritier J and Andlauer W 2012) |
|  | Epigallocatechin | 13.3 | C_15_H_14_O_7_ | 305.0671 | 261,0762; 169.0359; 125.0244 | (Wu C, Xu H, Héritier J and Andlauer W 2012) |
|  | Epicatechin | 17.6 | C_15_H_14_O_6_ | 289.0716 | 245.0823; 205.0500; 125.0239 | (Wu C, Xu H, Héritier J and Andlauer W 2012) |
|  | Epigallocatechin gallate | 21.9 | C_22_H_18_O_11_ | 457.0792 | 305.0681; 169.0154; 125.0249 | (Wu C, Xu H, Héritier J and Andlauer W 2012) |
|  | Gallocatechin gallate | 24.5 | C_22_H_18_O_11_ | 457.0794 | 305.0665; 169.0160; 125.0264 | (Wu C, Xu H, Héritier J and Andlauer W 2012) |
|  | Epicatechin gallate | 27.1 | C_22_H_18_O_10_ | 441.0886 | 289.0765; 169.0172; 125.0264 | (Wu C, Xu H, Héritier J and Andlauer W 2012) |
|  | Catechin gallate | 29.6 | C_22_H_18_O_10_ | 441.0854 | 289.0744; 169.0163; 125.0260 | (Wu C, Xu H, Héritier J and Andlauer W 2012) |
| S12 | Ferulic acid | 23.2 | C_10_H_10_O_4_ | 193.0537 | 134.0396 | METLIN (database) |
|  | 4-O-caffeoylquinic acid | 24.7 | C_16_H_18_O_9_ | 353.0926 | 191.0518; 179.0315; 173.0451; 135.0473 | (Clifford MN, Knight S, Surucu B and Kuhnert N 2006) |
|  | 5-O-caffeoylquinic acid | 25.9 | C_16_H_18_O_9_ | 353.0808 | 191.0518; 179.0316; 161.0233 | (Clifford MN, Knight S, Surucu B and Kuhnert N 2006) |
|  | 5-O-feruloylquinic acid | 34.6 | C_17_H_20_O_9_ | 367.1071 | 191.0591; 173.0477 | (Clifford MN, Knight S, Surucu B and Kuhnert N 2006) |
|  | Di-O-caffeoylquinic acid isomer | 51.9 | C_25_H_24_O_12_ | 515.1257 | 353.0910 | (Clifford MN, Knight S, Surucu B and Kuhnert N 2006) |
|  | Di-O-caffeoylquinic acid isomer | 53.3 | C_25_H_24_O_12_ | 515.1184 | 353.0083 | (Clifford MN, Knight S, Surucu B and Kuhnert N 2006) |
|  | Di-O-caffeoylquinic acid isomer | 54.1 | C_25_H_24_O_12_ | 515.1249 | 353.0930 | (Clifford MN, Knight S, Surucu B and Kuhnert N 2006) |
|  | O-dimethoxycinnamoyl, O-caffeoylquinic acid | 55.6 | C_27_H_28_O_12_ | 543.1517 | 381.1180; 335.0900 | (Jaiswal R et al. 2014) |
| S13 | Caffeoylquinic acid | 8.4 | C_16_H_18_O_9_ | 353.0863 | 191.0542; | (Clifford MN, Knight S, Surucu B and Kuhnert N 2006) |
|  | Quinic acid | 11.9 | C_7_H_10_O_6_ | 191.0590 | 85.0317 | METLIN (database) |
|  | Gallic acid | 22.4 | C_7_H_6_O_5_ | 169.0128 | 125.0232; 79.0188 | (Wu C, Xu H, Héritier J and Andlauer W 2012)Wu 2012 |
|  | Isoquercitrin | 28.9 | C_21_H_20_O_12_ | 463.0877 | 300.0238; 271.0247; 255.0295; 243.0284; 151.0051 | METLIN (database) |
|  | Rutin | 37.3 | C_27_H_30_O_16_ | 609.1425 | 300.0261 | METLIN (database) |
| S14 | Gallic acid | 2.6 | C_7_H_6_O_5_ | 169.0128 | 125.0232; 79.0188 | (Wu C, Xu H, Héritier J and Andlauer W 2012)Wu 2012 |
|  | Gallocatechin | 4.1 | C_15_H_14_O_7_ | 305.0671 | 261,0762; 169.0359; 125.0244 | (Wu C, Xu H, Héritier J and Andlauer W 2012)Wu 2012 |
|  | Epigallocatechin | 7.5 | C_15_H_14_O_7_ | 305.0671 | 261,0762; 169.0359; 125.0244 | (Wu C, Xu H, Héritier J and Andlauer W 2012)Wu 2012 |
|  | Epicatechin | 10.4 | C_15_H_14_O_6_ | 289.0716 | 245.0823; 205.0500; 125.0239 | (Wu C, Xu H, Héritier J and Andlauer W 2012)Wu 2012 |
|  | Catechin | 14.2 | C_15_H_14_O_6_ | 289.0712 | 245.0821; 205.0616; 125.0261 | (Wu C, Xu H, Héritier J and Andlauer W 2012)Wu 2012 |
|  | Epigallocatechin gallate | 18.8 | C_22_H_18_O_11_ | 457.0792 | 305.0681; 169.0154; 125.0249 | (Wu C, Xu H, Héritier J and Andlauer W 2012)Wu 2012 |
|  | Gallocatechin gallate | 19.7 | C_22_H_18_O_11_ | 457.0794 | 305.0665; 169.0160; 125.0264 | (Wu C, Xu H, Héritier J and Andlauer W 2012)Wu 2012 |
|  | Epicatechin gallate | 21.4 | C_22_H_18_O_10_ | 441.0886 | 289.0765; 169.0172; 125.0264 | (Wu C, Xu H, Héritier J and Andlauer W 2012)Wu 2012 |
|  | Catechin gallate | 22.9 | C_22_H_18_O_10_ | 441.0854 | 289.0744; 169.0163; 125.0260 | (Wu C, Xu H, Héritier J and Andlauer W 2012) |
|  | Quercitrin | 26.7 | C_21_H_20_O_11_ | 447.0979 | 301.0387 | (Sanchez-Rabaned F, O., Lamuela-Raventos RM, Bastida J, Viladomat F and Codina C 2003) |
|  | Myricetin | 27.1 | C_15_H_10_O_8_ | 317.9351 | 179.0953; 151.9542 | METLIN (database) |
|  | Resveratrol | 28.7 | C_14_H_12_O_3_ | 227.0716 | 185.0606; 143.0480 | METLIN (database) |
|  | Quercetin | 32.2 | C_15_H_10_O7 | 301.0373 | 178.9987; 151.0037 | (Sanchez-Rabaned F, O., Lamuela-Raventos RM, Bastida J, Viladomat F and Codina C 2003) |
|  | Methoxyquercetin | 36.6 | C_16_H_12_O_7_ | 315.0537 | 301.0321; 178.0952, 151.0040 | METLIN (database) |
|  | Kaempferol | 37.9 | C_15_H_10_O_7_ | 285.0014 | 151.0043 | METLIN (database) |
| S15 | Caffeic acid | 3.9 | C_9_H_8_O_4_ | 179.0363 | 135.0465 | METLIN (database) |
|  | Quinic acid | 4.7 | C_7_H_12_O_6_ | 191.0590 | 85.0317 | METLIN (database) |
|  | Caffeine | 8.2 | C_8_H_10_H_4_O_2_ | 193.0520 | 178.0253; 136.0455; 78.0636 | (Wu C, Xu H, Héritier J and Andlauer W 2012) |
|  | 4-O-caffeoylquinic acid | 8.8 | C_16_H_18_O_9_ | 353.0926 | 191.0518; 179.0315; 173.0451; 135.0473 | (Clifford MN, Knight S, Surucu B and Kuhnert N 2006) |
|  | Caffeoylquinic acid | 10.6 | C_16_H_18_O_9_ | 353.0863 | 191.0542; | (Clifford MN, Knight S, Surucu B and Kuhnert N 2006) |
|  | 5-O-caffeoylquinic acid | 13.9 | C_16_H_18_O_9_ | 353.0808 | 191.0518; 179.0316; 161.0233 | (Clifford MN, Knight S, Surucu B and Kuhnert N 2006) |
|  | Shikimic acid | 16.1 | C_7_H_10_O_5_ | 173.0470 | 135.0450; 111.0461 | METLIN (database) |
|  | 5-O-feruloylquinic acid | 18.0 | C_17_H_20_O_9_ | 367.1077 | 191.0580; 173.0473 | (Clifford MN, Knight S, Surucu B and Kuhnert N 2006) |
|  | Caffeoyl-1,5-quinolactone | 30.3 | C_16_H_16_O_8_ | 335.0800 | 161.0260; 135.0310 | METLIN (database) |
|  | Di-O-caffeoylquinic acid isomer | 28.5 | C_25_H_24_O_12_ | 515.1257 | 353.0912 | (Clifford MN, Knight S, Surucu B and Kuhnert N 2006) |
|  | Di-O-caffeoylquinic acid isomer | 30.2 | C_25_H_24_O_12_ | 515.1257 | 353.0911 | (Clifford MN, Knight S, Surucu B and Kuhnert N 2006) |
|  | Caffeoyl-1,5-quinolactone derivative | 30.9 | C_20_H_26_O_9_ | 409.1542 | 335.0800, 161.0260; 135.0310 | METLIN (database) |
|  | Di-O-caffeoylquinic acid isomer | 31.9 | C_25_H_24_O_12_ | 515.1257 | 353.0910 | (Clifford MN, Knight S, Surucu B and Kuhnert N 2006) |
|  | 3-O-feruloyl, 5-O-caffeoylquinic acid | 37.3 | C_19_H_30_O_17_ | 529.1356 | 367.1076; 335.0757; 179.0351; 161.0241; 135.0454 | (Clifford MN, Knight S, Surucu B and Kuhnert N 2006) |
| S16 | Phloroglucinol | 3.9 | C_6_H_6_O_3_ | 125.0268 | - | METLIN (database) |
|  | Epicatechin | 26.0 | C_15_H_14_O_6_ | 289.0716 | 245.0823; 205.0500; 125.0239 | (Wu C, Xu H, Héritier J and Andlauer W 2012) |
|  | Catechin | 32.1 | C_15_H_14_O_6_ | 289.0712 | 245.0821; 205.0616; 125.0261 | (Wu C, Xu H, Héritier J and Andlauer W 2012) |
|  | Epicatechin derivative | 36.4 | C_23_H_32_O_17_ | 579.1559 | 469.1191; 289.0753; 245.0857; 125.0246 | (Wu C, Xu H, Héritier J and Andlauer W 2012) |
|  | Epicatechin derivative | 38.2 | C_23_H_32_O_17_ | 579.1561 | 469.1185; 289.0756; 245.0857; 125.0248 | (Wu C, Xu H, Héritier J and Andlauer W 2012) |
|  | Epicatechin derivative | 40.4 | C_23_H_32_O_17_ | 579.1560 | 469.1189; 289.0750; 245.0853; 125.0245 | (Wu C, Xu H, Héritier J and Andlauer W 2012) |

References to the Table S2
