# Supplementary material for: LC-QTOF-MS Analysis and Activity Profiles of Popular Antioxidant Dietary Supplements in Terms of Quality Control
Source: Oxid Med Cell Longev. 2017 May 31;2017:8692516. doi: 10.1155/2017/8692516 (PMC5470020; doi:10.1155/2017/8692516)
Supplement: Supplementary file 2 [file 8692516.f2.pdf]

Supplementary material

Table S1. Antioxidant activity of dietary supplements.

	ORAC (Trolox equivalents (µM))			DPPH (Trolox equivalents (µM))			ABTS (Trolox equivalents (µM))			TPC (caffeic acid equivalents (mg))		
	1g of sample	Single dose	Dailydose	1g of sample	Single dose	Dailydose	1g of sample	Single dose	Dailydose	1g of sample	Single dose	Dailydose
**S1**	321.16	16.19	32.37	35.43	1.79	3.57	110.31	5.56	11.12	0.004645	0.23412	0.468
**S2**	709.27	60	120.01	888.61	75.18	150.35	1757.09	148.65	297.3	0.131354	11.11252	22.225
**S3**	2152.03	732.98	1465.97	1422.58	484.53	969.06	1707.5	581.57	1163.15	0.143195	48.7723	97.545
**S4**	562.02	130.73	130.73	186.53	43.39	43.39	401.35	93.35	93.35	0.017553	4.08292	4.083
**S5**	4225.85	758.12	758.12	2833.83	508.39	508.39	1865.96	334.75	334.75	0.365988	65.65827	65.658
**S6**	8603.54	81.73	81.73	2829.6	26.88	26.88	1823.88	17.33	17.33	0.342579	3.2545	3.255
**S7**	569.42	44.81	134.44	104.1	8.19	24.58	247.95	19.51	58.54	0.01614	1.27022	3.811
**S8**	142	32.25	32.25	7.71	1.75	1.75	31.43	7.14	7.14	0.000485	0.11013	0.11
**S9**	1510.08	169.28	169.28	919.57	103.08	103.08	1128.56	126.51	126.51	0.092319	10.34899	10.349
**S10**	341.72	60.48	362.9	13.21	2.34	14.03	62.92	11.14	66.82	0.004367	0.7729	4.637
**S11**	13814.24	2923.09	5846.19	9833.08	2080.68	4161.36	1664.31	352.17	704.33	0.706857	149.57097	299.142
**S12**	1567.7	594.32	594.32	194.53	73.75	73.75	557.07	211.19	211.19	0.034673	13.14436	13.144
**S13**	429.29	44.52	89.04	126.43	13.11	26.22	177.87	18.45	36.89	0.006789	0.70399	1.408
**S14**	5513.39	3431.54	3431.54	3484.45	2168.72	2168.72	1800.39	1120.56	1120.56	0.350137	217.92517	217.925
**S15**	6471.51	1851.5	3703	1647.46	471.34	942.68	1736.17	496.72	993.44	0.304618	87.15131	174.303
**S16**	2196.36	92.69	92.69	1410.6	59.53	59.53	1905.01	80.39	80.39	0.117284	4.94938	4.949

Table S2. The chemical composition of investigated dietary supplements

Sample	Compound	Rt (min)	Molecular formula	$[M-H]^-$ (m/z)	Fragment ions	Reference
S1	Curcumin	44.9	$C_{21}H_{20}O_6$	367.1172	217.0497; 173,0599; 149.0601; 134.0364	METLIN (database)
	Demethoxycurcumin	50.5	$C_{20}H_{18}O_5$	337.1051	217.0490; 173.0595; 149.0601; 119.0493	METLIN (database)
	Bisdemthoxycurcumin	51.5	$C_{19}H_{16}O_4$	307.0958	187.0393; 143.0497; 119.0501	METLIN (database)
S2	Dihydroxybenzaldehyde	14.5	$C_7H_6O_3$	137.0251	108.0220	METLIN (database)
	3-O-caffeoylquinic acid	17.0	$C_{16}H_{18}O_9$	353.0863	191.0542; 179.0328; 135.0435	(Clifford MN et al. 2006)
	5-O-caffeoylquinic acid	26.2	$C_{16}H_{18}O_9$	353.0808	191.0518; 179.0316; 161.0233	(Clifford MN, Knight S, Surucu B and Kuhnert N 2006)
	3-O-caffeoyl-1,5-quinolactone	37.1	$C_{16}H_{16}O_8$	335.0920	161.0690; 135.0440	METLIN (database)
	Rutin	49.8	$C_{27}H_{30}O_{16}$	609.1425	300.0261	METLIN (database)
S3	Epigallocatechin gallate	22.4	$C_{22}H_{18}O_{11}$	457.0792	305.0681; 169.0154; 125.0249	(Wu C et al. 2012)
	Gallic acid	25.2	$C_7H_6O_5$	169.0128	125.0232; 79.0188	(Wu C, Xu H, Héritier J and Andlauer W 2012)
	Epicatechin gallate	27.4	$C_{22}H_{18}O_{10}$	441.0886	289.0765; 169.0172; 125.0264	(Wu C, Xu H, Héritier J and Andlauer W 2012)
	Apigenin glucuronide isomer	29.4	$C_{21}H_{18}O_1$	445.0806	269.0488; 175.0296	(He L et al. 2016)
	Apigenin glucuronide isomer	32.0	$C_{21}H_{18}O_1$	445.0841	269.0514; 175.0282	(He L, Zhang Z, Lu L, Liu Y, Li S, Wang J, Song Z, Yan Z and Miao J 2016)
	Methoxy apigenin glucuronide isomer	32.9	$C_{22}H_{21}O_2$	459.1021	283.0647; 268.0352; 175.0274	METLIN (database)
	Apigenin glucuronide isomer	33.5	$C_{21}H_{18}O_1$	445.0841	269.0514; 175.0282	(He L, Zhang Z, Lu L, Liu Y, Li S, Wang J, Song Z, Yan Z and Miao J 2016)
	Methoxy apigenin glucuronide isomer	34.5	$C_{22}H_{21}O_2$	459.1021	283.0647; 268.0352; 175.0274	METLIN (database)
	Apigenin	44.1	$C_{15}H_{10}O_5$	269.0498	-	(He L, Zhang Z, Lu L, Liu Y, Li S, Wang J, Song Z, Yan Z and Miao J

						2016)
S4	Dihydroxybenzaldehyde	5.9	$C_7H_6O_3$	137.0251	108.0220	METLIN (database)
	Epicatechin	12.3	$C_{15}H_{14}O_6$	289.0716	245.0823; 205.0500; 125.0239	(Wu C, Xu H, Héritier J and Andlauer W 2012)
	Quinic acid	15.5	$C_7H_{12}O_6$	191.0590	85.0317	METLIN (database)
	Catechin	16.5	$C_{15}H_{14}O_6$	289.0712	245.0821; 205.0616; 125.0261	(Wu C, Xu H, Héritier J and Andlauer W 2012)
	Kaempferol hexoside	26.7	$C_{21}H_{20}O_{11}$	447.0960	285.0447	METLIN (database)
S5	Dihydroxybenzoic acid	4.7	$C_7H_6O_4$	153.0192	108.0228; 91.0204; 65.0019	METLIN (database)
	Dihydroxybenzaldehyde	6.2	$C_7H_6O_3$	137.0247	108.0222; 92.0266	METLIN (database)
	Epicatechin	12.5	$C_{15}H_{14}O_6$	289.0716	245.0823; 205.0500; 125.0239	(Wu C, Xu H, Héritier J and Andlauer W 2012)
	Catechin	32.1	$C_{15}H_{14}O_6$	289.0712	245.0821; 205.0616; 125.0261	(Wu C, Xu H, Héritier J and Andlauer W 2012)
	A-type procyanidin trimer	21.0	$C_{45}H_{36}O_{18}$	863.1839	711.1351; 575.1207; 423.0724; 287.0558; 165.0149	(Qiang L et al. 2015)
	Methyl gallate	23.1	$C_8H_8O_5$	183.0313	139.0379; 95.0570	METLIN (database)
	A-type procyanidin trimer	23.7	$C_{45}H_{36}O_{18}$	863.1839	711.1351; 575.1207; 423.0724; 287.0558; 165.0149	(Qiang L, Luo F, Zhao X, Liu Y, Hu G, Sun C, Li X and Chen K 2015)
	Proanthocyanidin A isomer	26.4	$C_{30}H_{24}O_{12}$	575.1048	539.0789; 449.0803; 289.0639; 245.0842; 125.0183	(Qiang L, Luo F, Zhao X, Liu Y, Hu G, Sun C, Li X and Chen K 2015)
	A-type procyanidin trimer	27.8	$C_{45}H_{36}O_{18}$	863.1839	711.1351; 575.1207; 423.0724; 287.0558; 165.0149	(Qiang L, Luo F, Zhao X, Liu Y, Hu G, Sun C, Li X and Chen K 2015)
	Proanthocyanidin A isomer	28.6	$C_{30}H_{24}O_{12}$	575.1048	539.0789; 449.0803; 289.0639; 245.0842; 125.0183	(Qiang L, Luo F, Zhao X, Liu Y, Hu G, Sun C, Li X and Chen K 2015)
	A-type procyanidin trimer	29.9	$C_{45}H_{36}O_{18}$	863.1839	711.1351; 575.1207; 423.0724; 287.0558; 165.0149	(Qiang L, Luo F, Zhao X, Liu Y, Hu G, Sun C, Li X and Chen K 2015)
	Resveratrol	37.9	$C_{14}H_{12}O_3$	227.0707	185.0595;143.0496	(Gao F et al. 2016)
	Quercetin	43.3	$C_{15}H_{10}O_7$	301.0373	178.9987; 151.0037	(Sanchez-Rabaned F et al. 2003)
S6	Hexose	1.5	$C_6H_{12}O_6$	179.0572	161.0490; 101.0224; 89.0295	METLIN (database)

	Gallic acid	2.6	$C_7H_6O_5$	169.0128	125.0232; 79.0188	(Wu C, Xu H, Héritier J and Andlauer W 2012)
	Epicatechin	12.5	$C_{15}H_{14}O_6$	289.0716	245.0823; 205.0500; 125.0239	(Wu C, Xu H, Héritier J and Andlauer W 2012)
	Piceid	26.6	$C_{20}H_{22}O_8$	389.1261	227.0720; 185.0553; 143.0514	METLIN (database)
	Catechin	31.7	$C_{15}H_{14}O_6$	289.0712	245.0821; 205.0616; 125.0261	(Wu C, Xu H, Héritier J and Andlauer W 2012)
	Resveratrol	37.9	$C_{14}H_{12}O_3$	227.0716	185.0606; 143.0480	(Gao F, Zhou T, Hu Y, Lan L, Heyden YV, Crommen J, Lu G and Fan G 2016)
	Resveratrol derivative	44.3	$C_{21}H_{30}O_{13}$	489.1588	227.0715; 185.0605	(Gao F, Zhou T, Hu Y, Lan L, Heyden YV, Crommen J, Lu G and Fan G 2016)
	Emodic acid	52.1	$C_{15}H_8O_7$	299.0173	255.0307; 227.0351; 211.0388; 183.0346; 155.0506	METLIN (database)
	Physcion	53.3	$C_{16}H_{12}O_5$	283.0615	268.0337; 240.0411	(Gao F, Zhou T, Hu Y, Lan L, Heyden YV, Crommen J, Lu G and Fan G 2016)
	Emodin	58.8	$C_{15}H_{10}O_5$	269.0458	240.0425; 225.0548; 210.0303; 197.0602; 182.0365; 171.0442	(Gao F, Zhou T, Hu Y, Lan L, Heyden YV, Crommen J, Lu G and Fan G 2016)
S7	Citric acid	1.7	$C_6H_8O_7$	191.0203	111.0087; 87.0103; 67.0216	METLIN (database)
	Dihydroxybenzoic acid	4.6	$C_7H_6O_4$	153.0197	108.0222, 91.0204; 65.0019	METLIN (database)
	Coumaric acid	6.8	$C_9H_8O_3$	163.0425	119.0481	METLIN (database)
	Quinic acid	10.8	$C_7H_{12}O_6$	191.0590	85.0317	METLIN (database)
	Shikimic acid	14.4	$C_7H_{10}O_5$	173.0456	137.8011; 111.0116; 93.0320	METLIN (database)
	Rutin	27.4	$C_{27}H_{30}O_{16}$	609.1425	300.0261	METLIN (database)
	Isoquercitrin	28.3	$C_{21}H_{20}O_{12}$	463.0877	300.0238; 271.0247; 255.0295; 243.0284; 151.0051	(Sanchez-Rabaned F, O., Lamuela-Raventos RM, Bastida J, Viladomat F and Codina C 2003)
S8	Maltose	1.9	$C_{12}H_{22}O_{11}$	341.1124	221.0693; 179.0583, 161.0478; 143.0371; 119.0367; 101.0264	METLIN (database)
	Maltohexaose	2.3	$C_{30}H_{52}O_{26}$	989.3267	827.2736; 665.2195; 503.1656; 341.1123; 161.0477	METLIN (database)
	Glucosyl hydroxycinnamate	15.0	$C_{15}H_{18}O_8$	325.0992	163.0429; 145.0344	(Bondia-Pons I et al. 2014)

	Coumaric acid	15.9	$C_9H_8O_3$	163.0425	119.0481	(Bondia-Pons I, Savolainen O, Törrönen R, Martinez JA, Poutanen K and Hanhineva K 2014)b
	Coumaric acid dihexoside	17.3	$C_{21}H_{28}O_{13}$	487.1506	325.0975; 163.0421; 119.0483	(Bondia-Pons I, Savolainen O, Törrönen R, Martinez JA, Poutanen K and Hanhineva K 2014)
	Coumaric acid hexoside	20.2	$C_{15}H_{18}O_8$	325.0975	163.0423; 119.0482	(Bondia-Pons I, Savolainen O, Törrönen R, Martinez JA, Poutanen K and Hanhineva K 2014)
	Feruloylquinic acid trihexoside	20.5	$C_{35}H_{50}O_{24}$	853.4157	691.3594; 529.3096	(Clifford MN, Knight S, Surucu B and Kuhnert N 2006)
	Citric acid	21.9	$C_6H_8O_7$	191.0580	111.0102; 87.0117; 67.0168	(Bondia-Pons I, Savolainen O, Törrönen R, Martinez JA, Poutanen K and Hanhineva K 2014)
	Rutin hexoside	33.0	$C_{33}H_{40}O_{21}$	771.2007	609.1506; 462.0858; 301.0379	(Bondia-Pons I, Savolainen O, Törrönen R, Martinez JA, Poutanen K and Hanhineva K 2014)
S9	HHDP-hexoside	1.9	$C_{20}H_{18}O_{14}$	481.0655	301.0017; 275.0251; 229.0046	(Brighenti V et al. 2016)
	HHDP-hexoside	2.2	$C_{20}H_{18}O_{14}$	481.0637	301.0012, 275.0231; 229.0051	(Brighenti V, Groothuis SF, Prencip FP, Amir R, Benvenuti S and Pellati F 2016)
	Gallagyl-hex (punicalin)	2.6	$C_{34}H_{22}O_{22}$	781.0568	601.0013; 448.9772	(Brighenti V, Groothuis SF, Prencip FP, Amir R, Benvenuti S and Pellati F 2016)
	Digalloyl-hexoside	5.4	$C_{20}H_{20}O_{14}$	483.0802	331.0703; 313.0586; 169.0167	(Brighenti V, Groothuis SF, Prencip FP, Amir R, Benvenuti S and Pellati F 2016)
	Gallic acid	11.8	$C_7H_6O_5$	169.0128	125.0232; 79.0188	(Wu C, Xu H, Héritier J and Andlauer W 2012)Wu 2012
	Ellagitannin	16.3	$C_{24}H_{14}O_{15}$	541.0369	301.026; 275.0221	METLIN (database)
	Ellagitannin	16.8	$C_{27}H_{22}O_{18}$	633.0744	463.0521; 301.0021, 275.0240	METLIN (database)
	Gallotannin	22.6	$C_{27}H_{24}O_{18}$	635.0903	483.0714; 331.0713; 169.0144	METLIN (database)
	Rutin	26.9	$C_{27}H_{30}O_{16}$	609.1425	300.0261	METLIN (database)

	Ellagic acid	30.1	$C_{14}H_6O_8$	300.0029	283.9937; 245.0045; 200.0136; 173.0249; 145.0305	METLIN (database)
S10	Unidentified	5.7	$C_{11}H_{11}N_2O_3$	218.0707	175.0635; 118.0289; 92.0287	-
S11	Gallic acid	4.0	$C_7H_6O_5$	169.0128	125.0232; 79.0188	(Wu C, Xu H, Héritier J and Andlauer W 2012)
	Gallocatechin	8.9	$C_{15}H_{14}O_7$	305.0671	261,0762; 169.0359; 125.0244	(Wu C, Xu H, Héritier J and Andlauer W 2012)
	Epigallocatechin	13.3	$C_{15}H_{14}O_7$	305.0671	261,0762; 169.0359; 125.0244	(Wu C, Xu H, Héritier J and Andlauer W 2012)
	Epicatechin	17.6	$C_{15}H_{14}O_6$	289.0716	245.0823; 205.0500; 125.0239	(Wu C, Xu H, Héritier J and Andlauer W 2012)
	Epigallocatechin gallate	21.9	$C_{22}H_{18}O_{11}$	457.0792	305.0681; 169.0154; 125.0249	(Wu C, Xu H, Héritier J and Andlauer W 2012)
	Gallocatechin gallate	24.5	$C_{22}H_{18}O_{11}$	457.0794	305.0665; 169.0160; 125.0264	(Wu C, Xu H, Héritier J and Andlauer W 2012)
	Epicatechin gallate	27.1	$C_{22}H_{18}O_{10}$	441.0886	289.0765; 169.0172; 125.0264	(Wu C, Xu H, Héritier J and Andlauer W 2012)
	Catechin gallate	29.6	$C_{22}H_{18}O_{10}$	441.0854	289.0744; 169.0163; 125.0260	(Wu C, Xu H, Héritier J and Andlauer W 2012)
S12	Ferulic acid	23.2	$C_{10}H_{10}O_4$	193.0537	134.0396	METLIN (database)
	4-O-caffeoylquinic acid	24.7	$C_{16}H_{18}O_9$	353.0926	191.0518; 179.0315; 173.0451; 135.0473	(Clifford MN, Knight S, Surucu B and Kuhnert N 2006)
	5-O-caffeoylquinic acid	25.9	$C_{16}H_{18}O_9$	353.0808	191.0518; 179.0316; 161.0233	(Clifford MN, Knight S, Surucu B and Kuhnert N 2006)
	5-O-feruloylquinic acid	34.6	$C_{17}H_{20}O_9$	367.1071	191.0591; 173.0477	(Clifford MN, Knight S, Surucu B and Kuhnert N 2006)
	Di-O-caffeoylquinic acid isomer	51.9	$C_{25}H_{24}O_{12}$	515.1257	353.0910	(Clifford MN, Knight S, Surucu B and Kuhnert N 2006)
	Di-O-caffeoylquinic acid isomer	53.3	$C_{25}H_{24}O_{12}$	515.1184	353.0083	(Clifford MN, Knight S, Surucu B and Kuhnert N 2006)
	Di-O-caffeoylquinic acid isomer	54.1	$C_{25}H_{24}O_{12}$	515.1249	353.0930	(Clifford MN, Knight S, Surucu B and Kuhnert N 2006)

	O-dimethoxycinnamoyl, O-caffeoylquinic acid	55.6	$C_{27}H_{28}O_{12}$	543.1517	381.1180; 335.0900	(Jaiswal R et al. 2014)
S13	Caffeoylquinic acid	8.4	$C_{16}H_{18}O_9$	353.0863	191.0542;	(Clifford MN, Knight S, Surucu B and Kuhnert N 2006)
	Quinic acid	11.9	$C_7H_{10}O_6$	191.0590	85.0317	METLIN (database)
	Gallic acid	22.4	$C_7H_6O_5$	169.0128	125.0232; 79.0188	(Wu C, Xu H, Héritier J and Andlauer W 2012)Wu 2012
	Isoquercitrin	28.9	$C_{21}H_{20}O_{12}$	463.0877	300.0238; 271.0247; 255.0295; 243.0284; 151.0051	METLIN (database)
	Rutin	37.3	$C_{27}H_{30}O_{16}$	609.1425	300.0261	METLIN (database)
S14	Gallic acid	2.6	$C_7H_6O_5$	169.0128	125.0232; 79.0188	(Wu C, Xu H, Héritier J and Andlauer W 2012)Wu 2012
	Gallocatechin	4.1	$C_{15}H_{14}O_7$	305.0671	261,0762; 169.0359; 125.0244	(Wu C, Xu H, Héritier J and Andlauer W 2012)Wu 2012
	Epigallocatechin	7.5	$C_{15}H_{14}O_7$	305.0671	261,0762; 169.0359; 125.0244	(Wu C, Xu H, Héritier J and Andlauer W 2012)Wu 2012
	Epicatechin	10.4	$C_{15}H_{14}O_6$	289.0716	245.0823; 205.0500; 125.0239	(Wu C, Xu H, Héritier J and Andlauer W 2012)Wu 2012
	Catechin	14.2	$C_{15}H_{14}O_6$	289.0712	245.0821; 205.0616; 125.0261	(Wu C, Xu H, Héritier J and Andlauer W 2012)Wu 2012
	Epigallocatechin gallate	18.8	$C_{22}H_{18}O_{11}$	457.0792	305.0681; 169.0154; 125.0249	(Wu C, Xu H, Héritier J and Andlauer W 2012)Wu 2012
	Gallocatechin gallate	19.7	$C_{22}H_{18}O_{11}$	457.0794	305.0665; 169.0160; 125.0264	(Wu C, Xu H, Héritier J and Andlauer W 2012)Wu 2012
	Epicatechin gallate	21.4	$C_{22}H_{18}O_{10}$	441.0886	289.0765; 169.0172; 125.0264	(Wu C, Xu H, Héritier J and Andlauer W 2012)Wu 2012
	Catechin gallate	22.9	$C_{22}H_{18}O_{10}$	441.0854	289.0744; 169.0163; 125.0260	(Wu C, Xu H, Héritier J and Andlauer W 2012)
	Quercitrin	26.7	$C_{21}H_{20}O_{11}$	447.0979	301.0387	(Sanchez-Rabaned F, O., Lamuela-Raventos RM, Bastida J, Viladomat F and Codina C 2003)
	Myricetin	27.1	$C_{15}H_{10}O_8$	317.9351	179.0953; 151.9542	METLIN (database)

	Resveratrol	28.7	$C_{14}H_{12}O_3$	227.0716	185.0606; 143.0480	METLIN (database)
	Quercetin	32.2	$C_{15}H_{10}O7$	301.0373	178.9987; 151.0037	(Sanchez-Rabaned F, O., Lamuela-Raventos RM, Bastida J, Viladomat F and Codina C 2003)
	Methoxyquercetin	36.6	$C_{16}H_{12}O_7$	315.0537	301.0321; 178.0952, 151.0040	METLIN (database)
	Kaempferol	37.9	$C_{15}H_{10}O_7$	285.0014	151.0043	METLIN (database)
S15	Caffeic acid	3.9	$C_9H_8O_4$	179.0363	135.0465	METLIN (database)
	Quinic acid	4.7	$C_7H_{12}O_6$	191.0590	85.0317	METLIN (database)
	Caffeine	8.2	$C_8H_{10}H_4O_2$	193.0520	178.0253; 136.0455; 78.0636	(Wu C, Xu H, Héritier J and Andlauer W 2012)
	4-O-caffeoylquinic acid	8.8	$C_{16}H_{18}O_9$	353.0926	191.0518; 179.0315; 173.0451; 135.0473	(Clifford MN, Knight S, Surucu B and Kuhnert N 2006)
	Caffeoylquinic acid	10.6	$C_{16}H_{18}O_9$	353.0863	191.0542;	(Clifford MN, Knight S, Surucu B and Kuhnert N 2006)
	5-O-caffeoylquinic acid	13.9	$C_{16}H_{18}O_9$	353.0808	191.0518; 179.0316; 161.0233	(Clifford MN, Knight S, Surucu B and Kuhnert N 2006)
	Shikimic acid	16.1	$C_7H_{10}O_5$	173.0470	135.0450; 111.0461	METLIN (database)
	5-O-feruloylquinic acid	18.0	$C_{17}H_{20}O_9$	367.1077	191.0580; 173.0473	(Clifford MN, Knight S, Surucu B and Kuhnert N 2006)
	Caffeoyl-1,5-quinolactone	30.3	$C_{16}H_{16}O_8$	335.0800	161.0260; 135.0310	METLIN (database)
	Di-O-caffeoylquinic acid isomer	28.5	$C_{25}H_{24}O_{12}$	515.1257	353.0912	(Clifford MN, Knight S, Surucu B and Kuhnert N 2006)
	Di-O-caffeoylquinic acid isomer	30.2	$C_{25}H_{24}O_{12}$	515.1257	353.0911	(Clifford MN, Knight S, Surucu B and Kuhnert N 2006)
	Caffeoyl-1,5-quinolactone derivative	30.9	$C_{20}H_{26}O_9$	409.1542	335.0800, 161.0260; 135.0310	METLIN (database)
	Di-O-caffeoylquinic acid isomer	31.9	$C_{25}H_{24}O_{12}$	515.1257	353.0910	(Clifford MN, Knight S, Surucu B and Kuhnert N 2006)
	3-O-feruloyl, 5-O-caffeoylquinic acid	37.3	$C_{19}H_{30}O_{17}$	529.1356	367.1076; 335.0757; 179.0351; 161.0241; 135.0454	(Clifford MN, Knight S, Surucu B and Kuhnert N 2006)
S16	Phloroglucinol	3.9	$C_6H_6O_3$	125.0268	-	METLIN (database)

Epicatechin	26.0	$C_{15}H_{14}O_6$	289.0716	245.0823; 205.0500; 125.0239	(Wu C, Xu H, Héritier J and Andlauer W 2012)
Catechin	32.1	$C_{15}H_{14}O_6$	289.0712	245.0821; 205.0616; 125.0261	(Wu C, Xu H, Héritier J and Andlauer W 2012)
Epicatechin derivative	36.4	$C_{23}H_{32}O_{17}$	579.1559	469.1191; 289.0753; 245.0857; 125.0246	(Wu C, Xu H, Héritier J and Andlauer W 2012)
Epicatechin derivative	38.2	$C_{23}H_{32}O_{17}$	579.1561	469.1185; 289.0756; 245.0857; 125.0248	(Wu C, Xu H, Héritier J and Andlauer W 2012)
Epicatechin derivative	40.4	$C_{23}H_{32}O_{17}$	579.1560	469.1189; 289.0750; 245.0853; 125.0245	(Wu C, Xu H, Héritier J and Andlauer W 2012)

References to the Table S2

Abd El-Baky HH, El Baz FK, El-Baroty GS. 2008. Characterization of nutraceutical compounds in blue green alga *Spirulina Maxima*. Journal of Medicinal Plants Research 10:292-300.

Bondia-Pons I, Savolainen O, Törrönen R, Martinez JA, Poutanen K, Hanhineva K. 2014. Metabolic profiling of Goji berry extracts for discrimination of geographical origin by non-targeted liquid chromatographycoupled to quadrupole time-of-flight mass spectrometry. Food Research International.63:132–138.

Braga MEM, Leal PF, Carvalho OE, Meireles MAA. 2003. Comparison of yield, composition, and antioxidant activity of turmeric (Curcuma longa L.) extracts obtained using various techniques. Journal of Agricultural and Food Chemistry.51:6604-6611.

Brighenti V, Groothuis SF, Prencip FP, Amir R, Benvenuti S, Pellati F. 2016. Metabolite fingerprinting of Punica granatum L. (pomegranate)polyphenols by means of high-performance liquid chromatographywith diode array and electrospray ionization-mass spectrometrydetection. Journal of Chromatography A.

Clifford MN, Knight S, Surucu B, Kuhnert N. 2006. Characterization by LC-MSn of Four New Classes of Chlorogenic Acids in Green Coffee Beans: Dimethoxycinnamoylquinic Acids, Diferuloylquinic Acids, Caffeoyl-dimethoxycinnamoylquinic Acids, and Feruloyl-dimethoxycinnamoylquinic Acids. Journal of Agricultural and Food Chemistry.54:1957-1969.

Database Name. Available from: https://metlin.scripps.edu/index.php.

Embuscado ME. 2015. Spices and herbs: Natural sources of antioxidants – a mini review. Journal of Functional Foods.18:811-819.

Gao F, Zhou T, Hu Y, Lan L, Heyden YV, Crommen J, Lu G, Fan G. 2016. Cyclodextrin-based ultrasonic-assisted microwave extraction and HPLC-PDA-ESI-IT-MSn separation and identification of hydrophilic andhydrophobic components of Polygonum cuspidatum: A green, rapid and effective process. Industrial Crops and Products.80:59–69.

Ghimire BK, Seong ES, Kim EH, Ghimeray AK, Yu ChY, Ghimire BK, Chung IM. 2011. A comparative evaluation of the antioxidant activity of some medicinal plants popularly used in Nepal. Journal of Medicinal Plants Research.10:1884-1891.

He L, Zhang Z, Lu L, Liu Y, Li S, Wang J, Song Z, Yan Z, Miao J. 2016. Rapid identification and quantitative analysis of the chemical constituents in Scutellaria indica L. by UHPLC–QTOF–MS and UHPLC–MS/MS. Journal Of Pharmaceutical and Biomedical Analysis 117:125-139.

Henning SM, Zhang Y, Rontoyanni VG, Huang J, Lee RP, Trang A, Nuernberger G, Heber D. 2014. Variability in the antioxidant activity of dietary supplements from pomegranate, milk thistle, green tea, grape seed, goji, and acai: Effects of in vitro digestion. Journal of Agricultural and Food Chemistry.62:4313-4321.

Huang D, Ou B, Hampsch-Woodill M, Flanagan J, Prior R. 2002. High-throughput assay of oxygen radical absorbance capacity (ORAC) using a multichannel liquid handling system coupled with a microplate fluorescence reader in 96-well format. Journal of Agricultural and Food Chemistry.50:4437-4444.

Jaiswal R, Febi Matei M, Subedi P, Kuhner N. 2014. Does roasted coffee contain chlorogenic acid lactones or/and cinnamoylshikimate esters? Food Research International.61:214–227.

Kratchanova M, Denev P, Ciz M, Lojek A, Mihailov A. 2010. Evaluation of antioxidant activity of medicinal plants containing polyphenol compounds. Comparison of two extraction systems. Acta Biochimica Polonica.2:229-234.

Madrigal-Carballo S, Rodriguez G, Krueger CG, Dreher M, Reed JD. 2009. Pomegranate (Punica granatum) supplements: Authenticity, antioxidant and polyphenol composition. Journal of Functional Foods.1:324-329.

Martin KR, Appel CL. 2010. Polyphenols as dietary supplements: A double-edged sword. Nutrition and Dietary Supplements.2:1-12.

Medina MB. 2011. Determination of the total phenolics in juices and superfruits by a novel chemical metod. Journal of Functional Foods.3:79-87.

Meléndez NP, Nevárez-Moorillón V, Rodríguez-Herrera R, Espinoza JC, Aguilar CN. 2014. A microassay for quantification of 2.2-diphenyl-1- picrylhydracyl (DPPH) free radical scavenging. African Journal of Biochemistry Research.8:14-18.

Murillo E, Britton GB, Durant AA. 2012. Antioxidant activity and polyphenol content in cultivated and wild edible fruits grown in Panama. Journal of Pharmacy And Bioallied Sciences.4:313-317.

Pérez D, Leighton F, Aspee A, Aliaga A, Lissi E. 2000. A comparison of methods employed to evaluate antioxidant capabilities. Biological Research.33:71-77.

Prior RL. 2015. Oxygen radical absorbance capacity (ORAC): New horizons in relating dietary antioxidants/bioactives and health benefits. Journal of Functional Foods.18:797-810.

Qiang L, Luo F, Zhao X, Liu Y, Hu G, Sun C, Li X, Chen K. 2015. Identification of Proanthocyanidins from Litchi (Litchi chinensis Sonn.) Pulp by LC-ESI-QTOF- MS and Their Antioxidant Activity. PLoS ONE.10.

Re R, Pellegrini N, Proteggente A, Pannala A, Yang M, Rice-Evans C. 1999. Antioxidant activity applying an improved ABTS radical cation decolorization assay. Free Radical Biology and Medicine.26:1231-1237.

Sanchez-Rabaned F, O. J, Lamuela-Raventos RM, Bastida J, Viladomat F, Codina C. 2003. Identification of phenolic compounds in artichoke waste by highperformance liquid chromatography–tandem mass spectrometry. Journal of Chromatography A.1008:57–72.

Schaich KM, Tian X, Xie J. 2015. Reprint of "Hurdles and pitfalls in measuring antioxidant efficacy: A critical evaluation of ABTS. DPPH. and ORAC assays". Journal of Functional Foods.18:782-796.

Seeram NP, Henning SM, Niu Y, Lee R, Scheuller HS, Heber D. 2006. Catechin and caffeine content of green tea dietary supplements and correlation with antioxidant capacity. Journal of Agricultural and Food Chemistry.54:1599-1603.

Shahidi F, Zhong Y. 2015. Measurement of antioxidant activity. . Journal of Functional Foods.18:757-781.

Silva EM, Souza JNS, Rogez H, Rees JF, Larondelle Y. 2007. Antioxidant activities and polyphenolic contents of fifteen selected plant species from the Amazonian region. Food Chemistry.101:1012-1018.

Singleton VL, Rossi JA. 1965. Colorimetry of total phenolics with phosphomolybdic– phosphotungstic acid reagents. The American Journal of Enology and Viticulture.16:144-158.

Stintzing F, Herbach K, Mosshammer M, Carle R, Yi W, Sellappan S, Akoh CC, Bunch R, Felker P. 2005. Color, betalain pattern, and antioxidant properties of cactus pear (Opuntia spp.) clones. Journal of Agricultural and Food Chemistry.53:442-451.

Tabart J, Kevers C, Pincemail J, Defraigne JO, Dommes J. 2009. Comparative antioxidant capacities of phenolic compounds measured by various tests. Food Chemistry.113:1226-1233.

Wang CC, Chu CY, Chu JO, Choy KW, Khaw KS, Rogers MS, Pang CP. 2004. Trolox-equivalent antioxidant capacity assay versus oxygen radical absorbance capacity assay in plasma. Clinical Chemistry.50:952-954.

Wu C, Xu H, Héritier J, Andlauer W. 2012. Determination of catechins and flavonol glycosides in Chinese tea varieties. Food Chemistry.132:144–149.

Wu X, Beecher GR, Holden JM, Haytowitz DB, Gebhardt SE, Prior RL. 2004. Lipophilic and Hydrophilic Antioxidant Capacities of Common Foods in the United State. Journal of Agricultural and Food Chemistry.52:4026-4037.

Zulueta A, Esteve MJ, Frígola A. 2009. ORAC and TEAC assays comparison to measure the antioxidant capacity of food products. Food Chemistry.114:310-316.
